# Regular Family Meals Associated with Nutritional Status, Food Consumption, and Sedentary and Eating Behaviors of Brazilian Schoolchildren and Their Caregivers

**DOI:** 10.3390/foods13233975

**Published:** 2024-12-09

**Authors:** Giovanna Angela Leonel Oliveira, Gabriela Buccini, Vivian S. S. Gonçalves, Muriel Bauermann Gubert, Natacha Toral

**Affiliations:** 1 Graduate Program in Human Nutrition, Faculty of Health Science, University of Brasilia, Brasília 70910-900, DF, Brazil; giovannaangela@gmail.com (G.A.L.O.); murielgubert@gmail.com (M.B.G.); 2 Department of Social and Behavioral Health, School of Public Health, University of Nevada, Las Vegas, NV 89154, USA; gabriela.buccini@unlv.edu; 3 Graduate Program in Public Health, Faculty of Health Science, University of Brasilia, Brasília 70910-900, DF, Brazil; vivian.goncalves@unb.br

**Keywords:** diet, food habits, nutritional status, child, parent

## Abstract

The influence of family meals on nutrition and health for families has been understudied, especially in low- and middle-income countries. We aimed to analyze associations between regular family meals and body mass index (BMI), food consumption, eating, and sedentary behaviors among Brazilian schoolchildren and their caregivers. A cross-sectional study was conducted with 1887 Brazilian schoolchildren aged 6–11 years and their caregivers. Caregivers provided sociodemographic data, their child’s weight and height, and the frequency of family meals. Schoolchildren provided data on gender and silhouette scale. Both provided their food consumption, eating, and sedentary behavior data. Associations between regular family meals and BMI, ultra-processed food consumption, and dietary diversity were assessed through multivariate logistic regression models. Correlations of regular family meals with eating and sedentary behaviors were assessed using Pearson chi-square. Regular family meals were frequent (86.6%), and they were associated with less unhealthy BMI in caregivers (AOR: 0.74; 95%CI: 0.5–0.9), as well as higher dietary diversity in caregivers (AOR: 1.66; 95%CI: 1.0–2.7) and in schoolchildren (AOR: 1.78; 95%CI: 1.4–2.3). The coexistence of high dietary diversity and both low ultra-processed food consumption (AOR: 1.45; 95%CI: 1.0–2.1) and healthy BMI (AOR: 1.41; 95%CI: 1.0–1.9) in children was associated with regular family meals. Regular family meals were correlated with healthy eating behaviors in child-caregiver dyads (*p* < 0.002) and with sedentary behavior in caregivers (*p* = 0.019). Our findings underscore regular family meals as a protective factor against malnutrition among Brazilian families.

## 1. Introduction

The double burden of malnutrition, i.e., the coexistence of both undernutrition and overweight, is more prevalent in low- and middle-income countries [1]. Malnutrition can manifest throughout the life cycle, affecting individuals, households, or populations across generations [1]. Specifically, malnutrition in childhood can lead to adverse health outcomes such as stunted growth [2] and cognitive impairment [3]. Therefore, addressing these adverse health outcomes due to malnutrition during early life is crucial, and it should consider the social determinants of health and healthy lifestyle choices such as practicing physical activity and adopting healthy eating behaviors [4,5].

Healthy eating behaviors include actions related to eating, such as how and in what way one eats [6], and are protective factors against malnutrition. The types and quantities of food consumed can be influenced by the situations and environmental context, including eating without distractions, calmly and regularly, and sharing meals with family [7]. Thus, the family environment strongly influences habits and behaviors associated with schoolchildren’s malnutrition [8].

A family meal is defined as a meal in which most, ideally all, immediate family members are present and eat together in the household [9]. These meals are associated with child/adolescent nutrition, nutritional status, risk behaviors, and well-being [10]. Research from high-income countries has demonstrated the positive impact of family meals on adolescents’ nutrition [11]. This practice is associated with increased consumption of fruits, vegetables, proteins, and calcium, and reduced consumption of sugary snacks and beverages [11]. Additionally, it promotes a more pleasant setting [7], family union [11], appetite self-regulation [11], and a lower BMI in children [12]. Research has shown that in schoolchildren, a higher frequency of family meals is associated with better mental health in both Japan [13] and Brazil [14]. A recent meta-analysis found a significant relationship between the frequency of meals and lower BMI in children [12].

In Brazil, the largest country in Latin America, three national studies have addressed family meals among adolescents aged 13–17 years [15,16]. The National Survey of School Health (PeNSE 2015) revealed that family meals five or more times per week were associated with a higher likelihood of frequent consumption of beans, fruits, and vegetables, as well as a lower likelihood of frequent consumption of snacks, salty ultra-processed foods, and fried snacks [15]. In the Brazilian Study of Cardiovascular Risks in Adolescents (ERICA 2013–2014), adolescents who had lunch and dinner with their caregivers almost every day or every day had a lower prevalence of obesity [16]. The third study, involving adolescents in ninth grade attending both public and private schools across Brazilian state capitals and the Federal District, demonstrated that having lunch or dinner with parents every day was associated with higher consumption of healthy foods [17].

The evidence of family mealtimes on health and psychosocial outcomes in adolescent populations globally [2] and in Brazil (Da Silva et al., 2020) is strong; however, there is a major gap in research focused on school-age children, specifically those between 6 and 11 years old, a period of developmental change when families are still the major influence on the emergence of new nutrition and health behaviors [18]. Providing nutrition and health behavioral knowledge during this age may have long-term implications, as it helps establish habits that can shape future behaviors [18].

Brazil has a population of 14,533,651 children enrolled in elementary schools [19], comprising 7.2% of the total population and ranging from 6 to 11 years of age. According to the 2023 Food and Nutrition Surveillance System, about a third (34.3%) of schoolchildren and 70.1% of adults have some form of underweight or overweight [20]. The high prevalence of ultra-processed food consumption, i.e., industrial formulations typically containing numerous ingredients [21], among schoolchildren (86%) and adults (73%) [22], is a significant contributor to the double burden of malnutrition in Brazil [23]. Therefore, a nationwide analysis focusing on school-age children, exploring the influence of family meals on nutritional status, ultra-processed food consumption, dietary diversity, and eating and sedentary behaviors in both schoolchildren and their caregivers, is critical to advance the understanding of the risk factors of the double burden of malnutrition in Brazil. Hence, this study aims to investigate the association between family meals and nutritional status, food consumption (including the coexistence of dietary diversity and ultra-processed food consumption), as well as eating and sedentary behaviors of Brazilian schoolchildren and their caregivers.

## 2. Materials and Methods

### 2.1. Study Design

An online nationwide cross-sectional survey included students from the first to the fifth grade of elementary schools in Brazil (ages 6–11), along with their respective primary caregivers. This survey is part of the Schoolchildren Nutrition Study (Estudo de Nutrição de Crianças Escolares—ENUCE in Portuguese) held in Brazil. The study was approved by the Research Ethics Committee of the School of Health Sciences at the University of Brasília (number 4,956,501). Caregivers consented to respond voluntarily to the ENUCE survey, and their children also consented through an Informed Assent Form. In addition, we adhered to the Strengthening the Reporting of Observational Studies in Epidemiology (STROBE) guidelines to report this study [24].

### 2.2. Sampling

The sample was calculated considering the following parameters: an estimated population of 14,533,651 children enrolled in the first to the fifth grades of elementary school in 2021 [19]; a prevalence of malnutrition (underweight and overweight) of 39.37% in Brazilian children aged 5–10 years according to the Food and Nutritional Surveillance System of Primary Healthcare in 2021 [22]; an absolute error of approximately 2.2%; and a confidence level of 95%. This resulted in a minimum sample of 1885 children. This sample was distributed in quotas, proportionally across the five Brazilian geographical regions (North, Northeast, Central-West, Southeast, and South) and by the type of school in which children were enrolled (public or private), according to the population data from the National School Census [19]. Additionally, a margin of 30% was considered for each quota, ensuring the adequacy of at least 70% of the population distribution, a technique adopted in other studies [25,26] (Table 1).

### 2.3. Inclusion and Exclusion Criteria

Inclusion criteria were children enrolled in the first to fifth grades of Brazilian elementary schools, along with their caregivers with internet access. The following individuals were excluded from the study: (1) children whose ages were below 6 or above 11 years; (2) children diagnosed with cognitive disorders; (3) children with disorders that could affect their dietary intake, such as food allergies, intolerances, and chronic or autoimmune conditions; (4) children with conditions that hindered anthropometric assessment; (5) participants who did not provide information about the frequency of family meals; (6) caregivers who could not report their own weight or height; and (7) pregnant caregivers.

### 2.4. Data Collection

Data collection was conducted through an online questionnaire. The 2023 Continuous National Household Sample Survey (PNAD Contínua) highlights a high level of internet accessibility in Brazil, particularly among students and urban households. According to the data, 91.9% of students reported using the internet within the last three months, and 95.3% of urban households had access to mobile networks [27]. However, it is important to acknowledge that not all households have internet access, which could introduce a degree of selection bias. While the results of the online questionnaire comprehensively represent the population with internet access, it is essential to clarify that they may not fully reflect the circumstances of the minority without connectivity.

Participants were recruited using the snowball technique, a sampling method in which existing participants refer or recruit new participants, creating a chain-like effect. This technique is often used when the target population is challenging to identify or reach directly [28]. However, a limitation of snowball sampling is its potential for selection bias, as it relies on participant referrals, which may lead to a non-representative sample and limit the generalizability of the findings. To address this limitation, the questionnaire remained open to answers until the minimum quotas for the five Brazilian geographical regions and the type of school were filled, from February to November 2022.

The questionnaire access link was disseminated through researchers’ and the university’s social media platforms, as well as through emails sent to educational and healthcare-related organizations: Councils of Nutritionists, Collaborative Centers for School Food and Nutrition, Unions of Municipal Education Directors, Municipal Education Departments, Confederations and Unions of Basic Education Workers, and private schools in Brazilian capitals.

The questionnaire underwent a pilot study for testing and adjustments. This pilot study was conducted in two private schools in a municipality in Goiás and one public school in the Federal District, selected based on researcher convenience.

Upon completing the questionnaire, participants were provided with the Brazilian Dietary Guidelines for the Population [29]. Additionally, individual results were sent via email to those participants who requested it, along with a booklet titled “How to Promote a Healthy Life for My Child? A Guide to Healthy Eating, Physical Activity, and Mental Health for Caregivers of Children age 6 to 10”, created by the researchers specifically for the survey.

### 2.5. Questionnaire

The online questionnaire was organized into two sections. The first was directed at caregivers, and the second one focused on and informed by children. All questions and scales used in this study have been validated for the Brazilian population and virtual self-assessment data collection [30].

The caregiver’s section included sociodemographic characteristics, the child’s and caregiver’s nutritional status, frequency of family meals, food consumption, and eating and sedentary behaviors. The sociodemographic data collected included their Brazilian geographical regions of residence (North, Northeast, South, Southeast, and Central-West), gender (male/female), and age (in years) of the child and the caregiver, as well as the educational level of the caregiver (categorized as elementary school, high school, and college degree or higher). Caregivers self-reported their weight (in kilograms) and height (in meters), as well as their child’s weight and height to provide nutritional status [31]. To enhance the reliability of parent-reported child anthropometric data, caregivers were asked if they knew the child’s weight and height. If the response was negative, the question was skipped. Caregivers’ prior day food consumption was assessed through a validated list of 12 markers of natural foods and 13 markers of ultra-processed foods [32,33,34]. To investigate caregivers’ eating behaviors, we used the validated scale developed to evaluate diet according to the recommendations of the Dietary Guidelines for the Brazilian Population. This scale encompasses dimensions related to meal planning (e.g., eating without distraction), eating model (e.g., meal regularity), food choices (e.g., type of food consumed), and domestic organization for meal preparation (e.g., participation in meal-related activities) [35]. For this study, four items from the scale that were more aligned with children’s eating behavior were selected: “*I try to eat slowly*” (planning dimension); “*I usually skip at least one of the main meals”* (eating model dimension); “*I usually eat sandwiches, savory, snacks, or pizza for lunch or dinner instead of freshly prepared dishes*” (food choice dimension); “*I usually engage in meal preparation at home*” (domestic organization dimension). For analysis purposes, “often” and “always” responses were coded as yes, while “never” and “rarely” responses were coded as no. The sedentary behavior of caregivers was assessed based on screen time on the previous day. Excessive sedentary behavior was defined for individuals reporting three or more hours per day watching television, using a computer, tablet, or mobile phone [32,36].

After completing the caregiver’s section, respondents were instructed that the child should continue to respond to the questionnaire without interference from the caregiver. 

The child’s section included their gender (male/female) and their perceived body image which was collected through the Body Silhouette Scale [30,37] (an 11-point series of silhouette figures ranging from very thin to very overweight), validated for Brazilian children aged 7–12 years [37]. This section also included the auto-informed child’s food consumption on the prior day, which was collected through the Illustrated Questionnaire of School Children’s Food Consumption (QUACEB) [38]. QUACEB is a children’s food consumption recall with images of 33 national and 10 regional foods, validated for Brazilian children aged 6–10 years [38]. The child’s eating and sedentary behaviors on the previous day were measured through the Illustrated Questionnaire of Eating and Sedentary Behaviors (QUICAS), an illustrated questionnaire used to assess eating and sedentary behaviors in children aged 7–10 years [39]. QUICAS investigates the presence of appropriate and inappropriate eating behaviors, including eating with distraction (planning dimension), meal regularity (eating model dimension), type of food consumed (food choice dimension), and participation in meal-related activities (domestic organization dimension). It also includes the frequency of screen use of five different electronic devices, such as a television, cell phone, tablet, computer, and video games. Following the recommendation of a maximum of 2 h of daily screen exposure for children aged 6–9 years [40,41,42,43], excessive sedentary behavior was estimated for the use of screens in more than one period (morning, afternoon, or evening) on the previous day, regardless of the type and quantity of devices.

### 2.6. Independent Variable

Family meals were defined as the main meals (lunch or dinner) eaten with the primary caregivers [15]. The frequency of family meals was assessed through the following question: “*Do you usually have lunch or dinner with the child participating in this study?*” Response options were—yes, every day; 5 to 6 days a week; 3 to 4 days a week; 1 to 2 days a week; rarely; or no. For this analysis, regular family meals included five or more days per week (reference category).

### 2.7. Outcomes

The outcomes included (1) nutritional status, (2) ultra-processed food consumption, (3) dietary diversity, and (4) eating and sedentary behavior.

(1) Nutritional Status: The nutritional status of both children and their caregivers was evaluated using body mass index (BMI). Multiple imputation by multinomial logistic regression was performed to handle data in which caregivers did not report the weight or height of the children (*n* = 439), which would represent a significant loss of data regarding the nutritional status of children (23.3%). This imputation was based on the child’s responses to the Body Silhouette Scale, gender, and date of birth [44,45,46].

The BMI was calculated as the ratio of weight in kg to the square of height in meters. Caregivers were classified as follows: underweight, with BMI <18.50 kg/m^2^; normal weight, with BMI between 18.50 kg/m^2^ and 24.99 kg/m^2^; and overweight/obese, with BMI equal to or greater than 25.0 kg/m^2^. Children had their nutritional status classified based on the BMI-for-age z-score, using AnthroPlus software v 1.0.4 [47]. Children were considered underweight if their BMI-for-age z-score was <−2; normal weight if the z-score was ≥−2 and ≤+1; and overweight if the z-score was >+1 [48]. The nutritional status of both caregivers and schoolchildren was classified as a binary variable: healthy BMI (reference group) or unhealthy BMI (i.e., underweight and overweight).

(2) Ultra-Processed Foods Score: The assessment of ultra-processed food consumption for both children and their caregivers used a score based on the original NOVA classification [20,49], designed to quantify the intake of ultra-processed foods [32,33]. The score ranges from 0 (indicating no consumption of ultra-processed foods) to 10 points, with each consumed ultra-processed food item contributing one point. For caregivers, the cutoff point to determine high consumption was set as proposed by the NOVA score [20,32], considering it reached when five or more of ten listed ultra-processed foods were consumed. For children, the score was divided into quintiles, with the first quintile representing low ultra-processed food consumption (used as the reference group) and the last quintile representing high consumption.
(3) Dietary Diversity: The categorization of the dietary diversity score for caregivers followed the proposal by FAO [50,51]. Caregivers’ dietary diversity scores were divided into quintiles, with those in the last quintile indicating high dietary diversity and those in the first quintile reflecting low dietary diversity. The cutoff point validated for high dietary diversity was based on a national study in Brazil [32], considering adults who consumed five or more groups of natural foods as protectors against chronic diseases (reference group).

The dietary diversity score for children adapted United Nations International Children’s Emergency Fund UNICEF’s proposal [52], considering eight groups of natural foods listed in the QUACEB and assigning one point for the consumption of each food item. The score ranges from zero (indicating no food consumed) to eight. The “breast milk” group in UNICEF’s proposal was replaced by a combination of the fruits and vegetables groups [52]. High dietary diversity was considered when five or more items from the list of eight natural foods were consumed [52]. The natural foods from QUACEB included in the dietary diversity score are detailed in Supplementary Materials Table S1.

(4) Eating and sedentary behaviors were classified as the presence or absence of these behaviors based on the response to the questionnaire.

### 2.8. Covariates

The sociodemographic covariates included were the Brazilian geographical region, the gender and age of the child and the caregiver, and the educational level of the caregiver, which was considered a proxy for socioeconomic status.

### 2.9. Data Analysis

**Figure 1:**
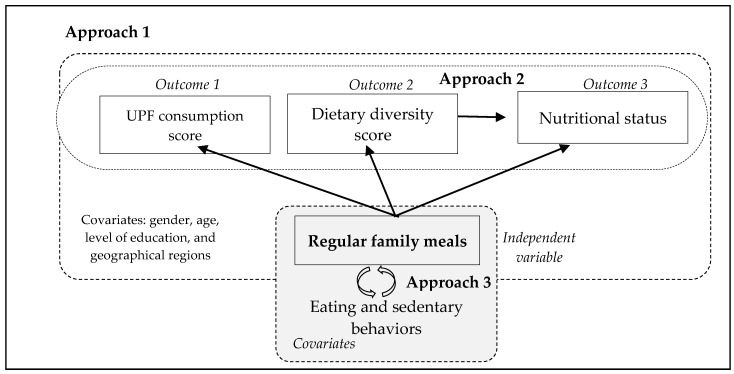
Theoretical model guiding the data analysis. Brazil, 2023. Legend: UPF: Ultra-Processed Food. Approach 1 (white color): Logistic regression analysis: exposure variable: frequency of family meals; outcomes: consumption of ultra-processed foods measured by the UPF consumption score, dietary diversity, and the nutritional status of children and their caregivers; adjustment covariates: gender, age, maternal educational level (proxy for socioeconomic status), and Brazilian geographical regions. Approach 2 (white color): Logistic regression analysis: exposure variable: frequency of family meals; outcomes: coexistence between consumption of ultra-processed foods measured by the UPF consumption score and dietary diversity; coexistence between the nutritional status and food consumption of both children and their caregivers; adjustment covariates: gender, age, maternal educational level (proxy for socioeconomic status), and Brazilian geographical regions. Approach 3 (gray color): Pearson’s chi-squared test between the variables “frequency of family meals” and dietary and sedentary behaviors of children and their caregivers.

Descriptive analyses explored differences in the outcome, independent variable, and covariates using absolute and relative frequencies, along with 95% confidence interval (95% CI). Figure 1 outlines a theoretical, analytical model illustrating the relationships between the outcomes, independent variables, and covariates that guided our analysis. All analyses were performed using the statistical software Stata, version 16.1. This study employed three analytical approaches to address the research questions, providing 95% confidence intervals for the results.

Approach 1 aimed to test the independent association between regular family meals and three outcomes—nutritional status, ultra-processed food consumption score, and dietary diversity score of children and their caregivers. Bivariate analyses were employed to examine the associations between the independent variable and outcomes. A *p*-value < 0.20 in the bivariate analysis served as the inclusion criterion for the adjusted multivariate model. Adjusted multivariate logistic regression models were conducted for each outcome, incorporating the independent variable and covariates.

Approach 2 aimed to explore factors co-occurring between food consumption and nutritional status associated with regular family meals. Identified risk factors for malnutrition included the simultaneous presence of (1) low dietary diversity combined with high ultra-processed food intake and (2) unhealthy BMI coupled with high ultra-processed food consumption. Conversely, protective factors for malnutrition were characterized by the simultaneous occurrence of (3) high dietary diversity with low ultra-processed food intake and (4) healthy BMI combined with high dietary diversity. Bivariate analyses were used to verify associations between the independent variables and outcomes of coexisting factors. A *p*-value < 0.20 in the bivariate analysis served as inclusion criteria for the adjusted multivariate model. Adjusted multivariate logistic regression models were conducted for each outcome, including the coexistence factors, independent variable, and covariates.

Approach 3 aimed to explore whether regular family meals are associated with eating and sedentary behaviors of children and their caregivers. Correlation was assessed using chi-square tests.

## 3. Results

A total of 1887 child-caregiver dyads participated in the survey. The sample was proportionally distributed across the type of school (public and private) and the five geographical regions of Brazil, with a margin of 30% of the population distribution. The comparison between the population and the sample distribution can be found in Supplementary Materials Table S2.

### 3.1. Descriptive Analysis

Table 2 outlines the descriptive analysis. A high proportion of caregivers reported having lunch or dinner with their children five days or more per week (86.59%), categorized as “regular family meals” in our study. Furthermore, children had an average consumption of three ultra-processed foods on the previous day (Standard Deviation (SD) = 2.0) and five natural foods (SD = 1.7). Caregivers had an average consumption of four ultra-processed foods (SD = 2.5) and six natural foods (SD = 1.8) on the previous day. It is noteworthy that a significant portion of both children (42.9%) and their caregivers (62.0%) were overweight/obese (Table 2).

The prevalence of children experiencing high dietary diversity and low consumption of ultra-processed foods (protective factors for malnutrition) was 19.5%, while 5.1% of children had low dietary diversity and high consumption of ultra-processed foods (risk factors for malnutrition). In contrast, 4.7% of caregivers presented both protective factors against malnutrition (high dietary diversity and low consumption of ultra-processed foods), and 13.3% presented both risk factors (low dietary diversity and high consumption of ultra-processed foods). One-third of the children (32.5%) simultaneously had healthy BMI and high dietary diversity, while 7.9% had unhealthy BMI and high consumption of ultra-processed foods. Conversely, 30.6% of the caregivers had an unhealthy BMI and high consumption of ultra-processed foods, while only 3.9% had a healthy BMI and high dietary diversity (Table 4).

### 3.2. Approach 1

Family meals occurring five days or more per week were associated with a decrease in the likelihood of the caregivers having an unhealthy BMI (AOR 0.74; 95%CI: 0.5–09), whereas no association with the schoolchild’s nutritional status was found (Table 3). Regular family meals were associated with an increased likelihood of high dietary diversity scores in both caregivers (AOR 1.66; 95%CI: 1.0–2.7) and children (AOR 1.78; 95%CI: 1.4–2.3) (Table 3). On the other hand, regular family meals were not associated with the consumption of ultra-processed foods in children and their caregivers (Table 3).

### 3.3. Approach 2

Regular family meals were associated with the coexistence of protective factors against malnutrition in children: high dietary diversity plus low ultra-processed food consumption (AOR 1.45; 95%CI: 1.0–2.1), and healthy BMI combined with high dietary diversity (AOR 1.41; 95%CI: 1.0–1.9). No statistically significant differences were found between regular family meals and the coexistence of protective factors for malnutrition among the caregivers and risk factors among both children and caregivers (Table 4).

### 3.4. Approach 3

Eating behaviors of both caregivers and schoolchildren were associated with regular family meals. They were positively correlated with caregivers who reported trying to eat slowly (*p* < 0.001), those engaged in meal preparation at home (*p* ≤ 0.001), those who did not usually skip main meals (*p* < 0.001), and those who opted for freshly prepared dishes over fast food or snacks (*p* ≤ 0.002). Regular family meals were also positively correlated with children who ate without distractions, ate at their usual times, ate “real” food, and participated in household activities involving meal preparation (*p* ≤ 0.001) (Table 5).

Regarding sedentary behaviors, regular family meals were associated with caregivers who spent less than 3 h a day using screens (*p* < 0.02). No association was found between regular family meals and children’s sedentary behavior (Table 5).

## 4. Discussion

Our study is innovative in assessing the influence of regular family meals on critical nutritional outcomes linked to the double burden of malnutrition in schoolchildren and their caregivers in Brazil. Regular family meals were associated with an increased likelihood of higher dietary diversity, the coexistence of protective factors against malnutrition such as high dietary diversity combined with low ultra-processed food consumption, healthy BMI coupled with high dietary diversity, and healthier eating behaviors in schoolchildren. In addition, regular family meals were associated with healthy BMI, higher dietary diversity, healthier eating behaviors, and reduced sedentary behaviors in caregivers. These findings can guide behavioral interventions focusing on reducing the double burden of malnutrition during a critical developmental stage to promote healthy lifestyles.

In our study, almost 87% of Brazilian caregivers and schoolchildren had lunch or dinner together five days or more per week. Prior evidence from a systematic umbrella review indicated that roughly between a quarter and half of families dined together several days a week, and about a third shared meals a few days a week [10]. Our findings documented a higher frequency of family meals during the COVID-19 pandemic phase when schools had just started to return to in-person activities compared to the systematic umbrella review conducted before the pandemic [10]. We hypothesized that the higher frequency found in our study may be due to changes in routine and family structure that contributed to the increase in family meals [53] during the lockdown. These changes may have persisted, creating new habits even after the partial return to in-person activities.

The positive association between regular family meals and better nutritional status among adults, but not among children, was surprising. Regular family meals have consistently been demonstrated to provide protection against obesity. However, not all studies endorse a solely positive impact of family meals, as increased family meal frequency may also correlate with higher energy intake and obesity [10,54]. Two potential explanations for the inconsistent findings regarding the correlation between family meal frequency and children’s nutritional status lie in sociodemographic characteristics and mealtime dynamics (i.e., meal type and family members present at the table) [12].

Our study did not find an association between regular family meals and a lower consumption of ultra-processed foods. This finding contrasts with a previous study conducted in Southern Brazil, where ultra-processed food consumption was less common during family meals but more prevalent on specific occasions, such as gatherings with family or friends, weekends, or when cooking was impractical due to other commitments [55]. Family meals are generally associated with lower consumption of UPFs due to the structured and home-cooked nature of these meals. However, certain contexts, social events, or weekends can influence the consumption of UPFs. We noted a high prevalence of ultra-processed food consumption among both children (16%) and their caregivers (45%), although our study did not assess on which occasions during the day the ultra-processed foods were consumed. Family meals that involve watching TV are associated with higher UPF consumption. This suggests that the quality of family meals may play a role in this association [56]. On the other hand, a recent study emphasized the influence of parents’ cooking skills as key to promoting healthy family meals and ultimately decreased ultra-processed food consumption [57]. Higher confidence in cooking from scratch is linked to a decrease in UPF consumption at dinner [57]. Parents’ cooking skills are influenced by sociocultural learning processes and the intergenerational transmission of cooking practices [58,59]. Additionally, involving children in food preparation can support healthy eating and cognitive development, while fostering a positive emotional climate and enhancing executive functioning skills [60].

In our study, regular family meals were associated with higher dietary diversity in schoolchildren and their caregivers. Corroborating our findings, caregivers and grand-caregivers in Taiwan who had regular family meals usually offered nutritious meals composed of local vegetables, suitable staples, and protein while also serving as role models for their school-age children [61]. Similar findings were found in Southern Brazil, where family meals are more likely to consist of whole foods, minimally processed items, and some processed products [55]. Therefore, there is evidence that regular family meals are associated with the provision of nutritious meals. Although our study did not directly assess the composition of family meals, the Dietary Guidelines for the Brazilian population suggest that sharing meals prepared at home generally leads to more nutritious choices compared to ultra-processed or ready-to-eat alternatives [7]. Homemade meals typically prioritize fresh ingredients, minimally processed foods, and a diverse range of food groups [7]. Our results underscore the potential effectiveness of promoting family meals in improving diet quality and health within households.

Regular family meals were associated with higher dietary diversity combined with lower consumption of ultra-processed foods or a combination of a healthy BMI with high dietary diversity. Corroborating our findings, a study conducted with Latino adolescents aged 10–14 years, regular family meals, in combination with positive food caregiver practices, were associated with a higher consumption of fruits [62]. In addition, several systematic reviews have indicated associations between regular family meals and healthier dietary patterns and nutrition status [10,12,54,63,64,65]. However, many families opt for processed foods at family meals to avoid conflicts and reduce stress at the table [66]. Purchasing fast food for family meals at least three times per week was positively associated with higher intake of salty snacks and soda among both parents and adolescents, as well as with increased weight status among parents. Conversely, limiting fast-food purchases for family meals to fewer than three times per week was associated with higher vegetable intake among parents [67].

Correlations were documented between regular family meals and eating behaviors in children and their caregivers, such as eating regularly and mindfully, eating in appropriate environments, and eating without distractions. Among Taiwanese schoolchildren, regular family meals were linked to not eating in front of the television and allowing everyone to focus on enjoying their meals [61]. In a municipality in Southern Brazil, the primary distractions found for schoolchildren during meals were watching TV (44.2%) and listening to music (20.9%) [55]. The authors note that these distractions also served as a backdrop for conversations at the table. Other activities carried out during meals, to a lesser extent, include family discussions (37.2%) or conversations about current events, news, and general updates (30.2%) [55]. Meals enjoyed around the table symbolize an important practice as a space for family conversations, the exchange of information, and dialogues, which also serve as a form of socialization within the household group. Furthermore, in addition to providing a conducive space for family interactions during meal consumption, family meals have been linked to increased participation in activities related to meal preparation. This underscores that family meals foster engagement in cooking practices, thereby promoting the preservation of culinary culture [68].

Regarding sedentary behavior, we found that regular family meals were associated with lower screen time for caregivers but not for schoolchildren. This finding suggests that other factors may influence children’s sedentary behavior or that the relationship between family meals and screen time for children is more complex than initially expected. In children aged 8–14 years, screen time served as a mediational factor in the relationship between family meals and depressive symptoms [69]. This implies a connection between family meals, screen time, and mental health in children, requiring further research for better understanding.

This study has strengths and limitations that should be considered when interpreting the results. First, our sample was collected using a snowball technique, which may result in selection bias. However, we employed a broad and stratified sampling approach, used in other studies [25,26], to ensure the most appropriate distribution of the population of children enrolled in the first to fifth grade of elementary schools (aged 6–11 years), considering different geographic regions and school types (public and private), which strengthened the national representation of our sample. Second, the data was collected through an online questionnaire, which may have restricted eligibility to only those with internet access. This requirement for internet access among caregivers limits the sample to those with such access. However, online surveys have been widely used in Brazil, as 90.0% of Brazilian households have internet access [27]. Additionally, parental reporting of children’s anthropometry and children reporting their dietary intake can differ significantly from actual measurements. On the one hand, this study reflects the COVID-19 pandemic period, which made direct measurement and information collection challenging. On the other hand, the ease of online data collection can be attributed to the hybrid period or the period following online classes when schools had increased contact with students’ caregivers. Nevertheless, our study involved a nationwide sample, which is useful for assessing large-scale trends across many variables related to nutritional status, food consumption, and behavioral habits, allowing for a comprehensive understanding of the factors associated with diet and health. Furthermore, by including both children and their caregivers, the study offers insights into the influences of family meals on both generations. Future studies should consider longitudinal measures to explore how family meals influence the physical and mental health of caregivers and children, as well as to explore the effectiveness of specific interventions to promote regular and healthy family meals.

## 5. Conclusions

In conclusion, the study indicates that regular family meals are associated with improved dietary diversity in schoolchildren and caregivers and a healthier BMI among caregivers (approach 1). Regular family meals are associated with the coexistence of protective factors against malnutrition in children (approach 2). Additionally, regular family meals are correlated with other protective eating behaviors in children and their caregivers, as well as with a less sedentary lifestyle for the caregivers (approach 3). These findings highlight the potential benefits of promoting regular family meals as a means of improving food consumption, eating behavior, and overall health in children and their caregivers in Brazil. Promoting regular family meals is crucial for better health, and it can be a strategic public health intervention when considering the household food environment.

## Figures and Tables

**Table 1:** Sample distribution according to the Brazilian geographical regions and type of school. Brazil, 2022.

Variables	Schoolchildren Population Estimated by School Census 2021 *n* (%)	Calculated Sample *n* (%)	Sample Adequacy for 30% Less or More	
			*n*	%
Brazil	14,533,651 (100)	1885 (100)	1320–2451	70.0–130.0
Geographical regions				
North	1,616,919 (11.12)	210 (11.12)	147–274	7.8–14.6
Northeast	4,132,922 (28.44)	536 (28.44)	375–697	19.9–36.9
Central-West	1,167,389 (8.03)	151 (8.03)	106–198	5.6–10.5
Southeast	5,660,515 (38.95)	734 (38.95)	514–954	27.3–50.6
South	1,955,906 (13.46)	254 (13.46)	178–330	9.4–17.5
Type of school				
Public	11,919,578 (82.01)	1546 (82.01)	1082–2010	57.4–106.6
Private	2,614,073 (17.99)	339 (17.99)	237–441	12.6–23.4

**Table 2:** Descriptive analysis of outcome variables: nutritional status and consumption of natural foods and ultra-processed foods by schoolchildren and their caregivers. Brazil, 2022.

Variables	Prevalence					
	Child			Caregiver		
	*n*	%	95%CI	*n*	%	95%CI
**Regular family meals (5 times or more/week)**						
Yes				1634	86.6	84.9–88.1
No				253	13.4	11.9–15.0
Outcomes						
Nutritional status (BMI)						
Healthy	1002	53.1	50.8–55.3	682	36.1	3.4–3.8
Unhealthy						
Underweight	75	4.0	3.1–4.9	35	1.9	1.3–2.6
Overweight/obese	810	42.9	40.7–45.2	1170	62.0	5.9–6.4
Ultra-processed food score						
Low	637	35.7	33.5–37.8	1048	55.5	53.3–57.8
High	300	15.9	14.3–17.6	839	44.5	42.2–46.7
Dietary diversity						
Low	762	40.4	38.2–42.6	299	15.8	14.3–17.6
High	1124	59.6	57.4–61.8	1588	84.2	82.4–85.7
High dietary diversity + low ultra-processed food						
No	1518	80.5	78.6–82.2	1798	95.3	94.2–96.1
Yes	369	19.5	17.8–21.4	89	4.7	3.8–5.7
Low dietary diversity + high ultra-processed food						
No	1791	94.9	93.8–95.8	1654	87.7	86.1–89.1
Yes	96	5.1	4.2–6.2	233	13.3	10.9–13.9
Healthy BMI + high dietary diversity						
No	1274	67.5	65.4–69.6	1812	96.1	95.0–96.8
Yes	613	32.5	30.4–34.6	75	3.9	3.2–4.9
Unhealthy BMI + high ultra-processed food						
No	1737	92.1	90.7–93.2	1310	69.4	67.3–71.4
Yes	150	7.9	6.8–9.2	577	30.6	28.5–32.7
Covariates						
Gender						
Male	974	51.6	49.4–53.9	118	6.3	5.2–7.4
Female	912	48.4	46.1–50.6	1766	93.7	92.5–94.7
Age (*n* = mean/% = Std. Dev.)	8	1.49	7.9–8.1	36	7.35	35.9–36.5
Educational level						
Elementary school				491	26.2	24.2–28.2
High school				810	43.2	40.9–45.4
Undergraduate or higher				575	30.6	28.6–32.8
Geographical region						
North				274	14.5	13.0–16.2
Northeast				464	24.6	22.7–26.6
Central-West				198	10.5	9.2–11.9
Southeast				667	35.4	33.2–37.5
South				284	15.0	13.5–16.7
Type of school						
Public	1636	86.7	85.1–88.2			
Private	254	13.3	11.8–14.9			

Legend: 95%CI: 95% confidence interval; BMI: Body mass index; Low consumption of ultra-processed food in child: 1st quintile; High consumption of ultra-processed food in child: 5th quintile; Low consumption of ultra-processed food in caregivers: four or fewer group; High consumption of ultra-processed food in caregiver: five or more group; Low dietary diversity in child: four or fewer group; High dietary diversity in child: five or more group; Low dietary diversity in caregiver: 1st quintile; High dietary diversity in caregiver: 5th quintile.

**Table 3:** Logistic regression analysis of regular family meals and their associations with nutritional status and food consumption of schoolchildren and their caregivers (outcome variables). Brazil, 2022.

Regular Family Meals (5 Times or More/Week)	Unhealthy BMI				High Dietary Diversity				High Ultra-Processed Food Scores			
	Child		Caregiver		Child		Caregiver		Child		Caregiver	
	OR (95%CI)	AOR ^a^ (95%CI)	OR (95%CI)	AOR ^a^ (95%CI)	OR (95%CI)	AOR ^a^ (95%CI)	OR (95%CI)	AOR ^a^ (95%CI)	OR (95%CI)	AOR ^a^ (95%CI)	OR (95%CI)	AOR ^a^ (95%CI)
Yes	0.79 (0.6–1.0)	0.79 (0.6–1.0)	0.76 (0.6–1.0)	0.74 ↓ (0.5–0.9) *	1.74 (1.3–2.3) **	1.78 ↑ (1.4–2.3) *	1.69 (1.0–2.7) *	1.66 ↑ (1.0–2.7) *	0.80 (0.6–1.1)	^b^	0.84 (0.6–1.1)	0.79 (0.6–1.0)
No	(ref)	(ref)	(ref)	(ref)	(ref)	(ref)	(ref)	(ref)	(ref)	(ref)	(ref)	(ref)

Legend: BMI: Body mass index; OR: Odds ratio; AOR: Adjusted odds ratio; 95%CI: 95% confidence interval. ^a^ Adjusted for age, gender, maternal education (a proxy for socioeconomic status), and Brazilian geographical region. ^b^ Models with *p* > 0.20 in the crude analysis were excluded from the adjusted analysis. * *p* < 0.05; ** *p* < 0.01. ↓ Negative association. ↑ Positive association.

**Table 4:** Logistic regression analysis between the frequency of family meals (exposure variable) and its associations with health and unhealthy patterns in children and their caregivers (outcome variables). Brazil, 2022.

	Protective Factors for Malnutrition								Risk Factors for Malnutrition							
Regular Family Meals	High Dietary Diversity + Low Ultra-Processed Food				Healthy BMI + High Dietary Diversity				Low Dietary Diversity + High Ultra-Processed Food				Unhealthy BMI + High Ultra-Processed Food			
	Child		Caregiver		Child		Caregiver		Child		Caregiver		Child		Caregiver	
	OR (95%CI)	AOR ^a^ (95%CI)	OR (95%CI)	AOR ^a^ (95%CI)	OR (95%CI)	AOR ^a^ (95%CI)	OR (95%CI)	AOR ^a^ (95%CI)	OR (95%CI)	AOR ^a^ (95%CI)	OR (95%CI)	AOR ^a^ (95%CI)	OR (95%CI)	AOR ^a^ (95%CI)	OR (95%CI)	AOR ^a^ (95%CI)
Yes	1.39 (0.9–1.9)	1.45 ↑ (1.0–2.1) *	1.60 (0.8–3.3)	1.61 (0.8–3.4)	1.39 (1.0–1.9)	1.41 ↑ (1.0–1.9) *	2.22 (0.9–5.5)	2.19 (0.9–5.5)	0.83 (0.5–1.5)	^b^	0.71 (0.5–1.0)	0.69 (0.5–10)	0.72 (0.4–1.1)	0.71 (0.4–1.1)	0.82 (0.6–1.0)	0.78 (0.6–1.0)
No	(ref)	(ref)	(ref)	(ref)	(ref)	(ref)	(ref)	(ref)	(ref)	(ref)	(ref)	(ref)	(ref)	(ref)	(ref)	(ref)

Legend: BMI: Body mass index; OR: Odds ratio; AOR: Adjusted odds ratio; 95%CI: 95% confidence interval; ref: reference. ^a^ Adjusted for age, gender, type of school, maternal education (a proxy for socioeconomic status), and Brazilian geographical region. ^b^ Models with *p* > 0.20 in the crude analysis were excluded from the adjusted analysis. * *p* < 0.05. ↑ Positive association.

**Table 5:** Association of regular family meals and sedentary/eating behaviors of schoolchildren and their caregivers. Brazil, 2022.

Sedentary and Eating Behaviors	Regular Family Meals						*p*-Value
	0–4 Days			5 or More			
	*n*	%	95%CI	*n*	%	95%CI	
Caregiver							
Planning: “*I try to eat slowly.*”							
No	105	41.5	35.5–47.7	460	28.2	26.0–30.4	<0.001 *
Yes	148	58.5	52.3–64.4	1174	71.8	69.6–73.9	
Eating modes: “*I usually skip at least one of the main meals.*”							
No	172	68.0	61.9–73.5	1356	82.9	81.0–84.7	<0.001 *
Yes	81	32.0	26.5–38.0	278	17.0	15.3–18.9	
Food choices: “*I usually take sandwiches, savory snacks, or pizza for lunch or dinner instead of freshly prepared dishes.*”							
No	188	74.3	68.5–79.3	1346	82.4	80.4–84.1	0.002 *
Yes	65	25.7	20.6–31.5	288	17.6	15.8–19.5	
Domestic organization: “*I usually engage in meal preparation at home.*”							
No	102	40.3	34.4–46.5	486	29.7	27.6–32.0	0.001 *
Yes	151	59.7	53.5–65.6	1148	70.3	67.9–72.4	
Sedentary behavior: using screen							
Less than 3 h	87	34.5	28.9–40.6	691	42.3	39.9–44.7	0.019 *
More than 3 h	165	65.5	59.4–71.1	941	57.7	55.2–60.0	
Child							
Eating behavior: “*eating with distractions*”							
Without watching television and using cell phones	109	43.1	37.1–49.3	890	54.5	52.0–56.9	0.001 *
Watching TV or using a cell phone	144	56.9	50.7–62.9	744	45.5	43.1–47.9	
Eating behavior: “*eating at regular times*”							
At the usual time	185	73.1	67.3–78.2	1423	87.1	85.4–88.6	<0.001 *
At a different time than usual	68	26.9	21.7–32.7	211	12.9	11.4–14.6	
Eating behavior: “*type of food*”							
Real food (rice, beans, beef, and salad)	230	90.9	86.7–93.9	1564	95.7	94.6–96.6	0.001 *
Fast or industrialized food	23	9.1	6.1–13.3	70	4.3	3.4–5.4	
Eating behavior: “*participation in household activities involving meal preparation.*”							<0.001 *
Yes	56	22.1	17.4–27.7	590	36.1	33.8–38.5	
No	197	77.9	72.3–82.6	1044	63.9	61.5–66.2	
Sedentary behavior: using screen							
Tolerable	56	22.1	17.4–27.7	410	25.1	23.0–27.2	0.310
Excessive	197	77.9	72.3–82.6	1224	74.9	72.7–76.9	

Legend: 95%CI: 95% confidence interval. * *p* < 0.02.

## Data Availability

The original contributions presented in the study are included in the article and Supplementary Material, further inquiries can be directed to the corresponding author.

## Supplementary Materials

The following supporting information can be downloaded at: https://www.mdpi.com/article/10.3390/foods13233975/s1, Table S1: Food groups for calculating the NOVA score and the dietary diversity score. Brazil, 2022; Table S2: Participants distributed according to the Brazilian macro-region and type of school. Brazil, 2022.
