# Supplementary material for: Regular Family Meals Associated with Nutritional Status, Food Consumption, and Sedentary and Eating Behaviors of Brazilian Schoolchildren and Their Caregivers
Source: Foods. 2024 Dec 9;13(23):3975. doi: 10.3390/foods13233975 (PMC11641401; doi:10.3390/foods13233975)
Supplement: Supplementary file 1 [file foods-13-03975-s001.zip › Table S1 suplementar_final.pdf]

Table S1. Food groups for calculating the NOVA score and the dietary diversity score. Brazil, 2022.

Score	Ultra-processed food groups		Natural or staple food groups	
	For children	For parents	For children	For parents
1	Soda	Soda	Rice, potato, or cassava/manioc	Rice, pasta, polenta, couscous, sweet corn, potato, cassava, taro, or yam
2	Industrialized juices in cartons	Fruit juice in a carton, box, or can, or powdered drink mix	Beans	Beans, peas, lentils, or chickpeas
3	Chocolate milk or flavored yogurt	Chocolate milk or flavored yogurt	Beef, pork, chicken, fish, or shrimp	Beef, pork, chicken, or fish
4	Packaged bread	Sliced bread, hot dog buns, or hamburger buns	Egg	Fried, boiled, or scrambled egg
5	Packaged Salty snacks or crackers	Packaged snacks (or chips) or savory biscuits/crackers	Milk	Milk
6	Cookie or packaged sweet cake	Sweet biscuits/cookies, filled biscuits/cookies, or packaged cakes	Squash, carrot, papaya, or mango	Pumpkin, carrot, sweet potato, okra/gumbo, papaya, mango, yellow melon, persimmon, or pequi
7	Chocolate, ice cream, gelatin, or candy	Chocolate, ice cream, jelly, pudding, or other processed desserts	Banana, apple, orange, tangerine, grape, or avocado	Orange, banana, apple, or pineapple
8	Salami, sausage, baloney, or ham	Sausage, chorizo, bologna, or ham	Broccoli, kale, tomato, chayote, cucumber, lettuce or cabbage	Kale, broccoli, watercress, or spinach
9	Margarine, mayonnaise, or ketchup	Margarine, mayonnaise, ketchup, or mustard		Tomato, cucumber, zucchini, eggplant, chayote, or beetroot
10	Instant noodles, frozen lasagna, or pizza	Instant noodles, packaged soup, frozen lasagna, or other purchased frozen ready meals		Peanut, cashew nut, or Brazil nut/Para nut

Source: compiled by authors.
