# Supplementary material for: Regular Family Meals Associated with Nutritional Status, Food Consumption, and Sedentary and Eating Behaviors of Brazilian Schoolchildren and Their Caregivers
Source: Foods. 2024 Dec 9;13(23):3975. doi: 10.3390/foods13233975 (PMC11641401; doi:10.3390/foods13233975)
Supplement: Supplementary file 1 [file foods-13-03975-s001.zip › Table S2 suplementar_final.pdf]

Table S2. Participants distributed according to the Brazilian macro-region and type of school. Brazil, 2022.

**Variables**	**Study sample**	**Distribution of the study sample**	**Divergence of the calculated sample**
	n	%	%
**Brazil**	1887	100.00	0
**Macro-Regions**			
North	274	14.52	+30
Northeast	464	24.59	-14
Central-West	198	10.49	+30
Southeast	667	35.35	-9
South	284	15.05	+12
**Type of school**			
Public	1636	86.70	+6
Private	254	13.30	-26

Source: compiled by authors.
